# Postsynaptic structure formation of human iPS cell-derived neurons takes longer than presynaptic formation during neural differentiation in vitro

**DOI:** 10.1186/s13041-021-00851-1

**Published:** 2021-10-11

**Authors:** Kazuyuki Togo, Hayato Fukusumi, Tomoko Shofuda, Hiroshi Ohnishi, Hiroyuki Yamazaki, Mariko Kato Hayashi, Nana Kawasaki, Nobuyuki Takei, Takanobu Nakazawa, Yumiko Saito, Kousuke Baba, Hitoshi Hashimoto, Yuko Sekino, Tomoaki Shirao, Hideki Mochizuki, Yonehiro Kanemura

**Affiliations:** 1 Department of Neurology, Graduate School of Medicine, Osaka University, Suita, Osaka 565-0871 Japan; 2 Division of Stem Cell Research, Department of Biomedical Research and Innovation, Institute for Clinical Research, National Hospital Organization Osaka National Hospital, Osaka, Osaka 540-0006 Japan; 3 Department of Laboratory Sciences, Gunma University Graduate School of Health Sciences, Maebashi, Gunma 371-8514 Japan; 4 Department of Neurobiology and Behavior, Gunma University Graduate School of Medicine, Maebashi, Gunma 371-8511 Japan; 5 Faculty of Social Welfare, Gunma University of Health and Welfare, Maebashi, Gunma 371-0823 Japan; 6 School of Medicine, International University of Health and Welfare, Narita, Chiba 286-8686 Japan; 7 Department of Food Science and Nutrition, Faculty of Food and Health Sciences, Showa Women’s University, Setagaya-ku, Tokyo 154-8533 Japan; 8 Laboratory of Biopharmaceutical and Regenerative Sciences, Graduate School of Medical Life Science, Yokohama City University, Yokohama, Kanagawa 230-0045 Japan; 9 Department of Brain Tumor Biology, Brain Research Institute, Niigata University, Niigata, Niigata 951-8585 Japan; 10 Laboratory of Molecular Neuropharmacology, Graduate School of Pharmaceutical Sciences, Osaka University, Suita, Osaka 565-0871 Japan; 11 Department of Bioscience, Faculty of Life Sciences, Tokyo University of Agriculture, Setagaya-ku, Tokyo 156-8502 Japan; 12 Graduate School of Integrated Sciences for Life, Hiroshima University, Higashi-Hiroshima, Hiroshima 739-8521 Japan; 13 Molecular Research Center for Children’s Mental Development, United Graduate School of Child Development, Osaka University, Suita, Osaka 565-0871 Japan; 14 Division of Bioscience, Institute for Datability Science, Osaka University, Suita, Osaka 565-0871 Japan; 15 Open and Transdisciplinary Research Initiatives, Osaka University, Suita, Osaka 565-0871 Japan; 16 Department of Molecular Pharmaceutical Sciences, Graduate School of Medicine, Osaka University, Suita, Osaka 565-0871 Japan; 17 Endowed Laboratory of Human Cell-Based Drug Discovery, Graduate School of Pharmaceutical Sciences, The University of Tokyo, Bunkyo-ku, Tokyo 113-0033 Japan; 18 Division of Regenerative Medicine, Department of Biomedical Research and Innovation, Institute for Clinical Research, National Hospital Organization Osaka National Hospital, 2-1-14 Hoenzaka, Chuo-ku, Osaka, Osaka 540-0006 Japan; 19 Department of Neurosurgery, National Hospital Organization Osaka National Hospital, Osaka, Osaka 540-0006 Japan

**Keywords:** Human-induced pluripotent stem cell, Neural progenitor cell, Vesicular glutamate transporter 2 (VGLUT2), Drebrin, PSD-95

## Abstract

The generation of mature synaptic structures using neurons differentiated from human-induced pluripotent stem cells (hiPSC-neurons) is expected to be applied to physiological studies of synapses in human cells and to pathological studies of diseases that cause abnormal synaptic function. Although it has been reported that synapses themselves change from an immature to a mature state as neurons mature, there are few reports that clearly show when and how human stem cell-derived neurons change to mature synaptic structures. This study was designed to elucidate the synapse formation process of hiPSC-neurons. We propagated hiPSC-derived neural progenitor cells (hiPSC-NPCs) that expressed localized markers of the ventral hindbrain as neurospheres by dual SMAD inhibition and then differentiated them into hiPSC-neurons in vitro. After 49 days of in vitro differentiation, hiPSC-neurons significantly expressed pre- and postsynaptic markers at both the transcript and protein levels. However, the expression of postsynaptic markers was lower than in normal human or normal rat brain tissues, and immunostaining analysis showed that it was relatively modest and was lower than that of presynaptic markers and that its localization in synaptic structures was insufficient. Neurophysiological analysis using a microelectrode array also revealed that no synaptic activity was generated on hiPSC-neurons at 49 days of differentiation. Analysis of subtype markers by immunostaining revealed that most hiPSC-neurons expressed vesicular glutamate transporter 2 (VGLUT2). The presence or absence of NGF, which is required for the survival of cholinergic neurons, had no effect on their cell fractionation. These results suggest that during the synaptogenesis of hiPSC-neurons, the formation of presynaptic structures is not the only requirement for the formation of postsynaptic structures and that the mRNA expression of postsynaptic markers does not correlate with the formation of their mature structures. Technically, we also confirmed a certain level of robustness and reproducibility of our neuronal differentiation method in a multicenter setting, which will be helpful for future research. Synapse formation with mature postsynaptic structures will remain an interesting issue for stem cell-derived neurons, and the present method can be used to obtain early and stable quality neuronal cultures from hiPSC-NPCs.

**Supplementary Information:**

The online version contains supplementary material available at 10.1186/s13041-021-00851-1.

## Background

Neural progenitor cells (NPCs) can be differentiated from human-induced pluripotent stem cells (hiPSCs) using various methods [1]; they are self-renewing and can differentiate into various types of neurons. Mature neurons induced from hiPSC-NPCs are useful for the physiological, pathological, and pharmacological characterization of human neurons because their accessibility is not limited and because they are free from ethical issues regarding the preparation of neuronal cultures from the human brain [2–6]. With this background, in vitro application of stem cell-derived neurons has been expected, and methods to induce differentiation into various types of neurons have been developed. Stem cell-derived neurons are also expected to be applied to synapses, which transmit information between neurons. There are many neurological diseases that are believed to be related to synaptic dysfunction, such as Alzheimer's disease, Huntington's disease, and Parkinson's disease [7].

In neural tissue, a substantial amount of excitatory synaptic transmission is mediated by glutamate. Vesicular glutamate transporters carry glutamate to synaptic vesicles, and three isoforms have been identified in mammals, including humans: vesicular glutamate transporter 1 (VGLUT1), vesicular glutamate transporter 2 (VGLUT2), and vesicular glutamate transporter 3 (VGLUT3). To date, hiPSC-NPCs have been reported to terminally differentiate into neurons such as VGLUT1-positive glutamatergic neurons and glutamate decarboxylase 67 (GAD67)-positive GABAergic neurons [8–11]. However, there have been a few reports on the induction of excitatory neurons other than VGLUT1-positive glutamatergic neurons. Although it has been reported that synapses themselves change from immature to mature states as neurons mature, few papers have clearly demonstrated when human stem cell-derived neurons change to mature synaptic structures [12]. Several reports have shown that hiPSC-derived neurons (hiPSC-neurons) have neurophysiological activity in vitro [13], but only a few reports have confirmed the localization of mature synaptic markers such as the PSD-95 protein. Moreover, most previous studies used mainly the direct induction method without a continuous proliferation process for NPCs, and the types of neurons that differentiate from in vitro expanded NPCs (which can advantageously be induced into a relatively homogeneous neuronal population on a large scale) and the extent of synapse-related molecule maturity remain unclear [8, 14].

In this study, we differentiated hiPSC-NPCs that were established using dual SMAD inhibition and expanded them by neurosphere culture with epidermal growth factor (EGF), fibroblast growth factor 2 (FGF2), and leukemia inhibitory factor (LIF). We examined the expression profiles of neuronal lineage, presynaptic, and postsynaptic markers. We hypothesized that some time would be required to allow for the expression and localization of PSD-95 in human-derived cells. To capture the changes in the synaptic structure at an earlier stage, we evaluated a marker called drebrin. There are two subtypes of drebrin in mammals, including in humans, drebrin A and drebrin E [15, 16]. This marker is an actin-binding protein that replaces drebrin E with drebrin A by alternative splicing during neuronal maturation [17, 18], and we also evaluated the effect of nerve growth factor (NGF) on the synaptic maturation of hiPSC-NPCs. NGF is produced in various parts of the CNS, and its action is particularly important for the survival of cholinergic nerves in the basal forebrain and has been reported to be involved in dendrite formation, at least in cholinergic neurons [19–21]. Thus, we expected that the presence or absence of NGF would alter synaptogenesis and differentiation maturation into cholinergic nerve cells. We believe that this study will clarify the in vitro process of neurogenesis and synapse formation of hiPSC-NPCs and provide useful information about the potential for future in vitro neuronal research and pharmacological testing applications using these hiPSC-neurons.

## Materials and methods

### Ethics statement

This study was conducted in accordance with the principles of the Declaration of Helsinki, and the use of hiPSCs and human fetal neural tissue-derived neural stem/progenitor cells (hN-NSPCs) was approved by the ethics committee of Osaka National Hospital (#110, 120, and 146).

### Cell lines

We used two hiPSC lines, 1210B2 and 1201C1 [22, 23], which were established from peripheral mononuclear blood cells using an integration-free reprogramming method and a feeder-free protocol (1210B2) [24] or SNL feeder cells (1201C1) [25]. Both lines were induced into NPCs using the dual SMAD inhibition method with dorsomorphin (FUJIFILM Wako Pure Chemical Corporation, Osaka, Japan) or LDN-193189 (Axon Medchem, Groningen, Netherlands) plus SB431542 (Sigma-Aldrich, St. Louis, MO, USA), as previously described [22, 23]. These hiPSC-NPCs were propagated using the neurosphere method in DMEM/Ham’s F-12 (DMEM/F12; FUJIFILM Wako Pure Chemical Corporation) supplemented with EGF (20 ng/mL; PeproTech, Rocky Hill, NJ, USA), FGF2 (20 ng/mL; PeproTech), LIF (10 ng/mL; Millipore, Billerica, MA, USA), B27 Supplement (B27, 2%; Thermo Fisher Scientific, Waltham, MA, USA), and heparin (1/1000 dilution; AY Pharmaceuticals, Tokyo, Japan) [26]. The medium was changed every 3–5 days. The cells were passaged every 10–12 days using TrypLE Select CTS (Thermo Fisher Scientific) at 37 °C for 5 min for single-cell dissociation and then resuspended in DMEM/Ham’s F-12 medium containing B27 Supplement, heparin, LIF, EGF, and FGF2, at a density of 1 × 10^5^ cells/mL [27].

Frozen dissociated primary hippocampal neurons of rats (SKY neurons; AlzMed, Tokyo, Japan) were cultured in PLO-coated dishes for 21 days according to the manufacturer's instructions and used as controls for immunostaining and electrophysiological analysis [28, 29].

### In vitro neuronal differentiation

**Fig. 1:**
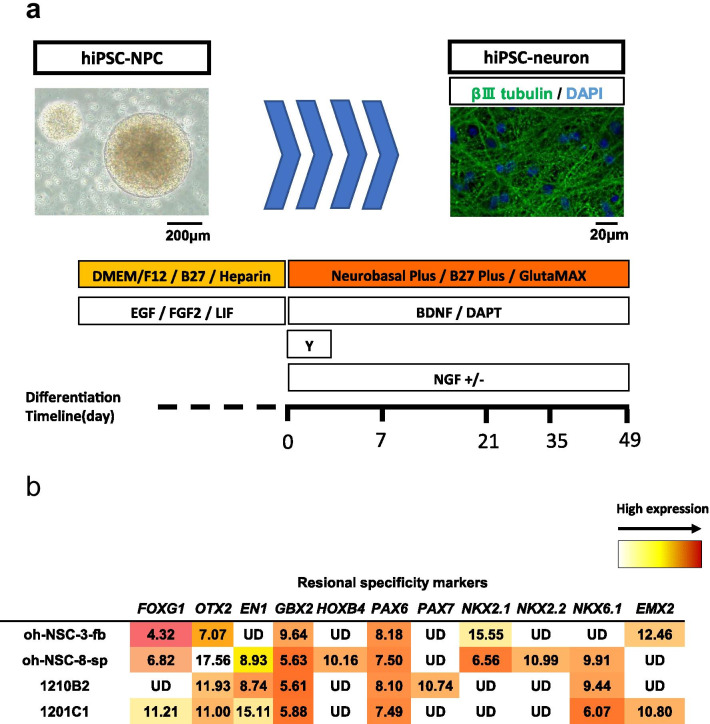
Procedure for the neuronal differentiation and characterization of hiPSC-NPCs. **a** Procedure for the neuronal differentiation of hiPSC-NPCs. The phase-contrast image shows the neurosphere of the hiPSC-NPCs before differentiation, and the immunofluorescence image shows neurons derived from hiPSC-NPCs (green: βIII-tubulin, blue: DAPI). Scale bars: 200 μm and 20 μm. **b** Gene expression of hiPSC-NPCs (1210B2 and 1201C1) and human neural tissue-derived NSPCs (oh-NSC-3-fb and oh-NSC-8-sp) before differentiation. The ΔCt values are shown. Red: high expression. *UD* undetermined

In vitro neuronal induction was performed as shown in Fig. 1a. Neurospheres were dissociated into single cells and seeded onto poly-l-ornithine (0.1 mg/mL; Sigma-Aldrich)-coated 96-well plates (ibidi, Gräfelfing, Germany) at a density of 2.25 × 10^5^ cells/cm^2^ in Neurobasal Plus Medium (Thermo Fisher Scientific) containing B27 Plus Supplement (B27 Plus, 2%; Thermo Fisher Scientific), GlutaMAX (0.5 mM; Thermo Fisher Scientific), gamma-secretase inhibitor, DAPT (10 µM; Abcam, Cambridge, UK), and brain-derived neurotrophic factor (BDNF, 20 ng/mL; PeproTech). The Rho-associated coiled-coil forming kinase inhibitor Y-27632 (Y, 10 µM; FUJIFILM Wako Pure Chemical Corporation) was added only on the 1st day. One day later (day 1 of differentiation), the medium was exchanged (half of the volume), and half of the medium was exchanged every 3–4 days thereafter. The culture was continued until day 49 of differentiation in the presence or absence of nerve growth factor (10 ng/mL; PeproTech).

### Quantitative reverse transcription-polymerase chain reaction (qRT-PCR)

The total RNA was separated using QIAzol Lysis Reagent (QIAGEN, Hilden, Germany), and cDNA was synthesized using the PrimeScript RT Reagent Kit (Takara Bio Inc., Shiga, Japan) according to the manufacturer’s instructions. qRT-PCR analysis was performed using gene-specific primers (Additional file 1: Table 1), PowerUp SYBR Green Master Mix (Thermo Fisher Scientific), and the 7300 Real-Time PCR System (Applied Biosystems, Foster City, California, USA), and the relative expression levels were calculated by the comparative threshold cycle (Ct) method.

Four control RNAs were used: two from hN-NSPCs (oh-NSC-3-fb and oh-NSC-8-sp) [26], human brain cerebellum total RNA and human brain cerebrum total RNA (Takara Bio Inc., CA, USA).

### Immunocytochemical staining

The cells were fixed with 4% paraformaldehyde phosphate buffer solution (FUJIFILM Wako Pure Chemical Corporation) at room temperature, washed three times with phosphate-buffered saline (PBS), and then blocked with a blocking solution (10% normal goat serum, 0.1% Triton X-100, PBS) for 30–60 min at room temperature. The blocking solution was then replaced with the primary antibody solution shown in Additional file 2: Table 2. After incubation at 4 °C overnight, the primary antibody solution was removed, and the cells were washed three times with PBS. Next, the cells were incubated with an AlexaFluor 488-conjugated goat anti-mouse IgG antibody (Thermo Fisher Scientific), an AlexaFluor 568-conjugated goat anti-rabbit IgG antibody (Thermo Fisher Scientific), and DAPI (Dojindo, Kumamoto, Japan) for 1 h at room temperature. The stained samples were then evaluated with a confocal laser scanning microscope (LSM700; Carl Zeiss, Hallbergmoos, Germany).

Under each condition, the number of ELAVL3/4-positive cells among 150 or more DAPI-positive cells was determined. In addition, ten visual fields were examined for each staining condition, and the proportions of cells that were positive for VGLUT1, VGLUT2, tyrosine hydroxylase (TH), tryptophan hydroxylase 2 (TPH2), vesicular acetylcholine transporter (VAChT), choline acetyltransferase (ChAT), and glial fibrillary acidic protein (GFAP) were determined.

### Western blot analysis

The cells were lysed with cell lysis buffer (2% SDS, 50 mM Tris–HCl pH 8.0, 150 mM NaCl), and 30 µg of protein from each sample was separated using sodium dodecyl sulfate–polyacrylamide gel electrophoresis (SDS-PAGE) and then electrophoretically transferred to polyvinylidene difluoride membranes (GE healthcare, Chicago, IL, USA). The membranes were blocked with 2% ECL Prime Blocking Reagent (cat. RPN418, GE healthcare) and incubated at 4 °C overnight with the primary antibody solution (Additional file 3: Table 3). After washing with Tris-buffered saline with Tween 20 (TBS-T), the membranes were incubated with secondary antibodies against rabbit or mouse horseradish peroxidase-conjugated immunoglobulin G (IgG, Cell Signaling Technology). Protein bands were detected by ECL Prime Western Blotting Detection Reagents (cat. RPN2232; GE healthcare), and images were obtained with the LAS4000 (Fujifilm, Tokyo, Japan). For reprobing, the membrane was stripped with stripping buffer (cat. T7135A; Takara Bio), blocked with 2% ECL Prime Blocking Reagent, and reprobed with different primary and secondary antibodies. Blotting with an anti-β-actin antibody (Additional file 3: Table 3) was used as the control. Quantification was performed with ImageJ software (Fiji package; National Institutes of Health, Bethesda, MD, USA). Ten micrograms of rat cerebrum tissue lysate (Takara Bio) was loaded and used as the control.

### Microelectrode array (MEA) recording

MEA recording was performed using the 60-channel MEA2100 system (Multi Chanel Systems, Reutlingen, Germany). The hiPSC-NPCs were differentiated into 6-well plates using the same methods as above, and hiPSC-neurons on day 49 were prepared for MEA recording. Spontaneous activity measurements were performed at 37 °C at a sampling rate of 20 kHz and filtered using a 200–3000 Hz band-pass filter. After measurement in differentiation medium without added glutamate, the whole volume of the medium was changed to differentiation medium with 100 μM glutamate, and the measurement was performed again. The obtained waveforms were analyzed using NeuroExplorer Version 5.306 (Nex Technologies, Madison, Alabama, USA). The standard deviation (SD) was calculated, and − 6.0 × SD of the baseline electrode noise was set as the negative threshold [30]. The negative peaks that exceeded the threshold were calculated as spikes and the presence of bursts was also analyzed by MaxInterval method [31]. SKY neurons at 21 days in vitro were used as a positive control, and wells with no cells were seeded as a negative control.

### Statistical analysis

The ΔCt value was calculated based on normalization to glyceraldehyde-3-phosphate dehydrogenase (GAPDH) for each condition. For each condition, the average ΔCt value was calculated, and a box plot was created. The ΔCt value of *drebrin A* based on *drebrin A*+*E* was calculated to determine the fractional ratio of drebrin. Student’s *t*-tests and one-way analysis of variance (ANOVA) were used for ΔCt values obtained under each qRT-PCR condition. When a significant difference was found in the ANOVA tests, multiple comparison tests using the Tukey–Kramer method were performed as post hoc analysis. A p-value < 0.05 was considered to be statistically significant. Data that could not be reproduced were treated as missing values.

### Multicenter validation study

Cells from the 1210B2 line were transported in a cryopreserved form and subsequently used. Then, the cells were cultured in DMEM/Ham’s F-12 medium containing B27 Supplement, heparin, LIF, EGF, and FGF2 for 10 days and dispersed into single cells with Neuron Dissociation Solutions S (FUJIFILM Wako Pure Chemical Corporation) according to the manufacturer’s instructions. Then, the hiPSC-NPCs were differentiated into neurons using the same induction method as that used in the absence of NGF described above. Total RNA was extracted before induction and at 49 days after neuronal differentiation using an RNeasy plus micro kit (QIAGEN), and RNA extraction samples from each facility were collected. Then, cDNA synthesis was performed using Prime Script RT (Takara Bio Inc.). qRT-PCR analysis was performed as described above using the comparative threshold cycle (Ct) method. The ΔCt values calibrated by normalization to *GAPDH* were determined for each gene, and the high and low expression levels were analyzed using a heat map to examine the robustness of our differentiation induction method.

## Results

### Expression of the ventral hindbrain marker in hiPSC-NPCs

We first characterized the hiPSC-NPCs just before neuronal differentiation. Regional markers along the anterior/posterior and dorsal/ventral axes of both the 1210B2 and 1201C1 cell lines were examined using qRT-PCR. Human fetal forebrain-derived NSPCs (oh-NSC-3-fb) and fetal spinal cord-derived NSPCs (oh-NSC-8-sp) were used as controls. Both hiPSC-NPC lines expressed the ventral hindbrain markers *GBX2* (mid/hindbrain) and *NKX6-1* at relatively high levels (Fig. 1b), showing that the two hiPSC-NPCs have hindbrain regional specificity. Focusing on the expression of other localization markers, *FOXG1*, a forebrain marker, was highly expressed in oh-NSC-3-fb cells. *OTX2*, a forebrain and midbrain marker, was also relatively expressed in oh-NSC-3-fb cells, while these markers were not expressed well in the hiPS-NPCs used in this study. *HOXB4*, a marker of spinal cord localization, was expressed in oh-NSC-8-sp cells but not in the oh-NSC-3-fb, 1210B2 or 1201C1 cell lines.

### Gene expression of pre- and postsynaptic markers increased during neuronal differentiation

**Fig. 2:**
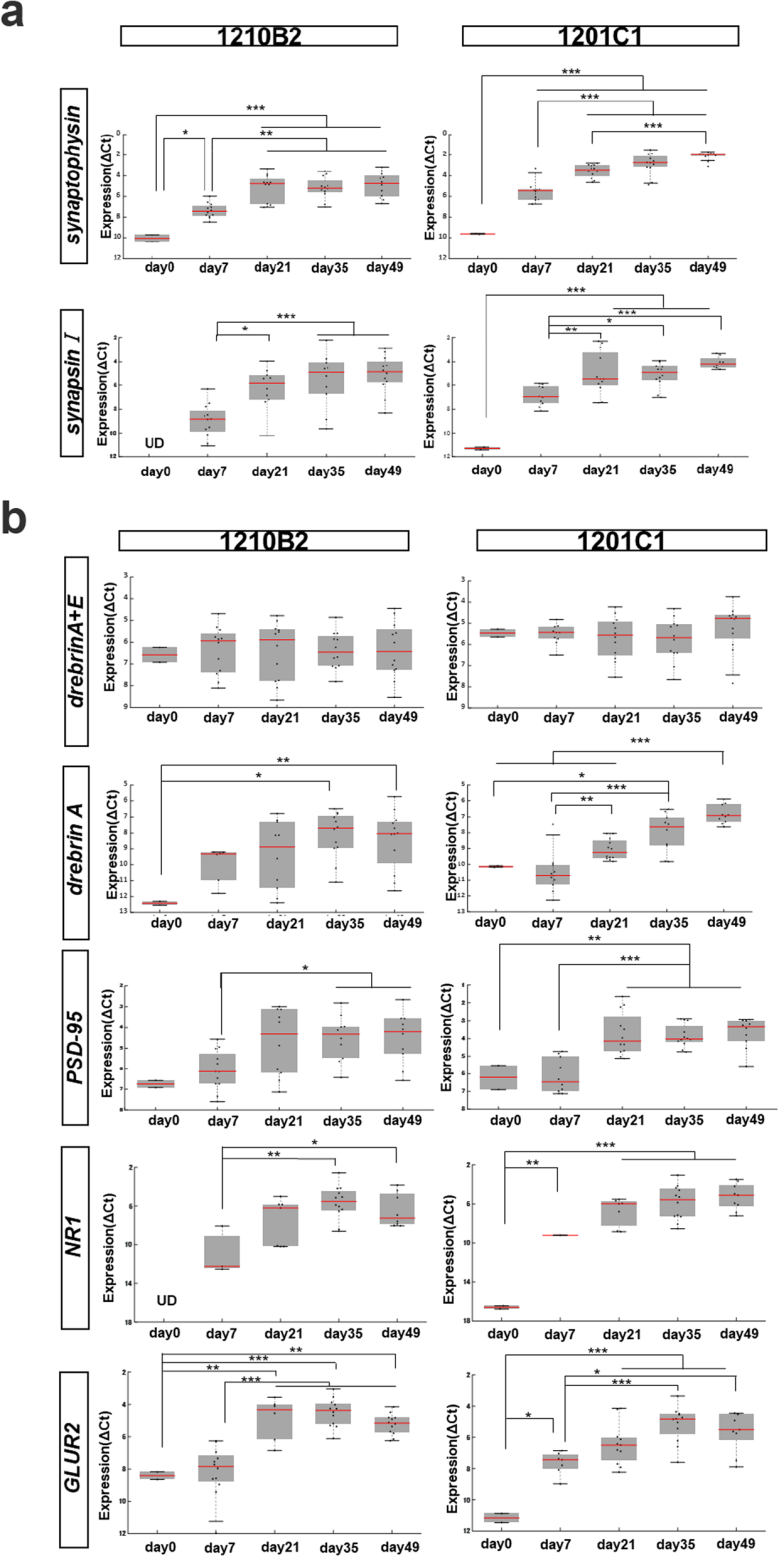
Gene expression levels of pre- and postsynaptic markers during the neuronal differentiation of hiPSC-NPCs. Dot plots and boxplots showing the expression of **a** presynaptic markers (*synaptophysin* and *synapsin I*) and **b** postsynaptic markers (*drebrin A*+*E*, *drebrin A*, *PSD-95, NR1, GLUR2*) during neuronal differentiation. Statistical analysis was performed using ANOVA with the post hoc Tukey–Kramer method (***p < 0.001, **p < 0.01, *p < 0.05). *UD* undetermined

mRNA transcription analysis by qRT-PCR demonstrated that the expression levels of *synaptophysin (SYP)* and *synapsin I (SYN1)* (presynaptic markers) as well as *drebrin A*, *PSD-95*, *NR1* and *GLUR2* (postsynaptic markers) significantly increased in the two hiPSC-NPC lines as neuronal differentiation progressed (Fig. 2a, b). Moreover, the expression level of *drebrin A*+*E* did not change during differentiation (Fig. 2b). We also examined the effect of NGF on the gene expression of *drebrin A*+*E*, *drebrin A*, and *SYP*; however, there were no significant differences in the expression levels of the three genes between the groups with and without NGF (Additional file 4: Fig. S1). These findings indicated that hiPSC-NPC terminal differentiation was accompanied by the expression of pre- and postsynaptic marker genes and the isoform conversion from *drebrin E* to *drebrin A* and that NGF had no significant effect on the expression of pre- and postsynaptic marker genes or on the isoform conversion of drebrin.

### Protein expression of pre- and postsynaptic markers in hiPSC-neurons on day 49 of differentiation

**Fig. 3:**
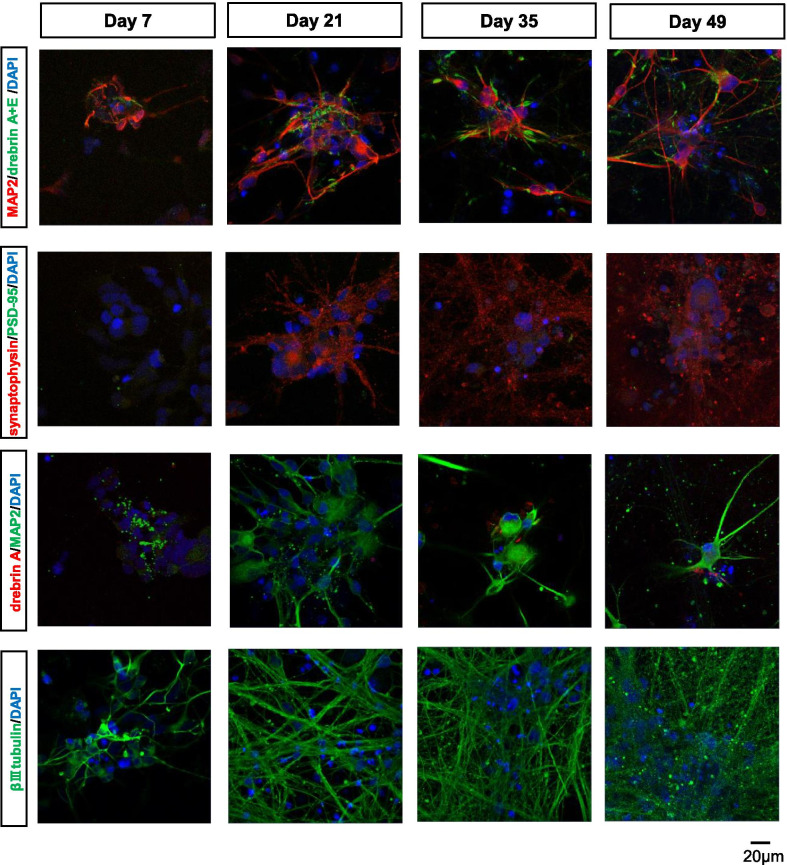
Protein expression of postsynaptic markers in terminally differentiated 1201C1 hiPSC-neurons. Time-dependent immunofluorescence images of βIII-tubulin, MAP2, total drebrin, drebrin A, synaptophysin, and PSD-95 in 1201C1 cells without NGF. The nuclei were stained with DAPI (blue). Scale bar: 20 μm

Immunofluorescence microscopy showed marked levels of βIII-tubulin-positive axonal growth in hiPSC-neurons during the differentiation period (Fig. 3 and Additional file 4: Fig. S2). Both microtubule-associated protein 2 (MAP2)-positive dendrites and pan-drebrin (drebrin A+E)-positive dendrites were obviously expressed during the differentiation period (Fig. 3 and Additional file 4: Fig. S2). A presynaptic marker, synaptophysin (SYP), was clearly expressed on day 21 of differentiation, and its expression level increased over time. However, the expression of a postsynaptic marker, drebrin A, was weak, even after day 35. Additionally, the expression of another postsynaptic marker, PSD-95, was poor even on day 49 in comparison with sufficient expression on day 21 SKY neurons (Fig. 3, Additional file 4: Figs. S2 and S3). As with transcript expression, NGF did not show a significant effect on the expression of synapse-related proteins (Additional file 4: Fig. S4). These findings suggested that the hiPSC-neurons expressed neuronal markers and synapse-related proteins at the protein level. However, the expression levels of these proteins were different; the expression of postsynaptic markers was relatively modest and lower than that of presynaptic markers.

**Fig. 4:**
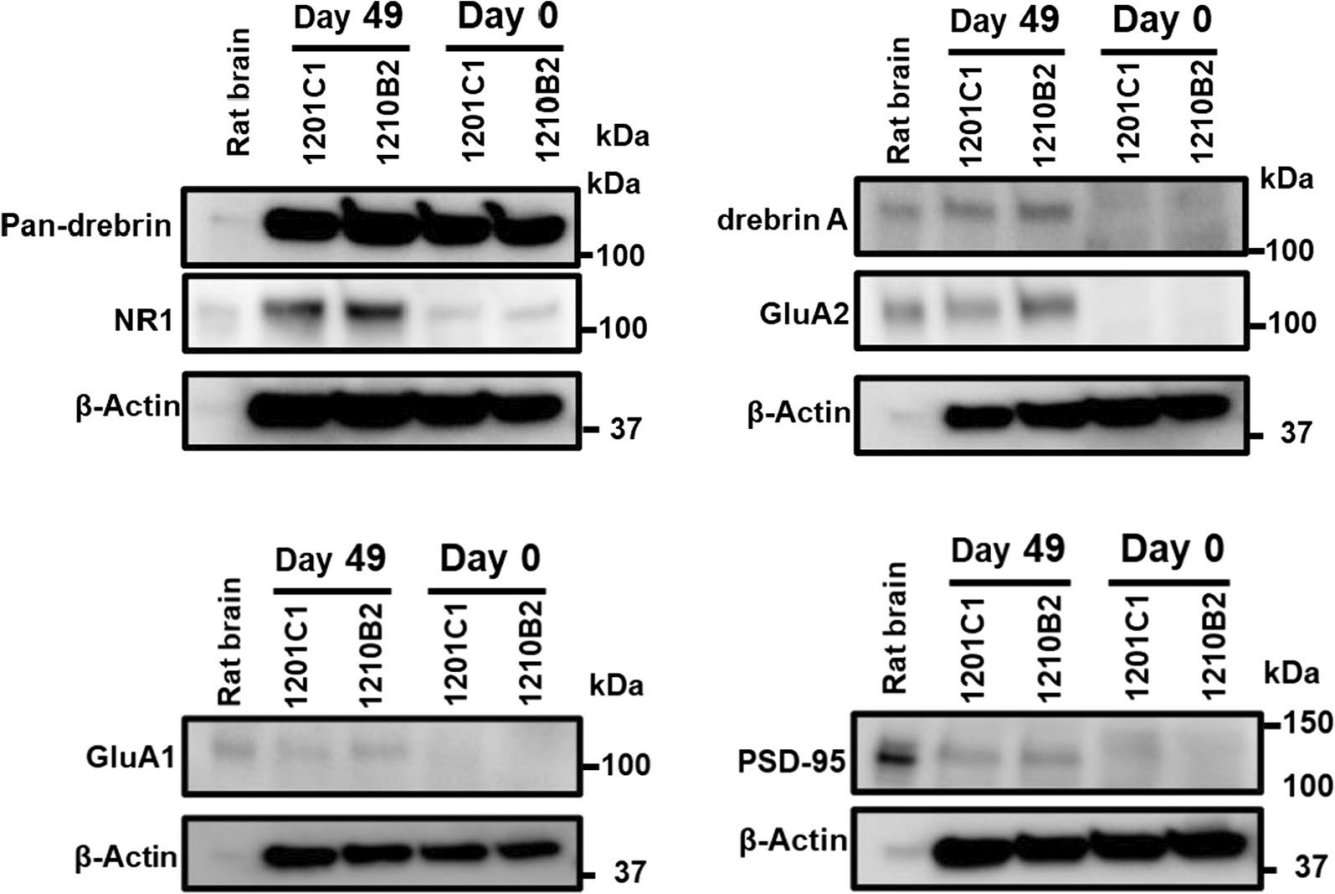
Western blot analysis of postsynaptic proteins in hiPSC neurons on day 0, day 49 and rat cerebrum tissue lysate. For the rat cerebrum tissue sample, 10 µg of protein per well was used, and for the other samples, 30 µg of protein per well was used for analysis

The expression levels of pre- and postsynaptic markers were also validated using Western blotting (Additional file 4: Fig. S5 and Fig. 4). Presynaptic markers, βIII-tubulin and synaptophysin, were both expressed much higher in hiPSC-neurons (day 49) than hiPSC-NPCs (day 0); however, their relative expression levels were lower than those in rat cerebral tissues (Additional file 4: Figs. S5, S6). The pan-drebrin was expressed equally in both hiPSC-NPCs (day 0) and hiPSC-neurons (day 49), and semiquantitative analysis showed that their expression levels were almost stable and slightly higher than those in rat cerebral tissues, which was consistent with the results of mRNA analysis (Additional file 4: Fig. S6). Drebrin A was significantly detected only in hiPSC-neurons (day 49); however, its relative expression level was much lower than that in rat cerebral tissues (Fig. 4 and Additional file 4: Fig. S6). Other postsynaptic markers, including PSD-95, GluA1 and GluA2, were expressed at detectable levels only in hiPSC-neurons, but their expression levels were also lower than those in rat cerebral tissues (Fig. 4 and Additional file 4: Fig. S6). NR1 was expressed in hiPSC-NPCs and increased after neuronal differentiation, but it remained at approximately one tenth of the level in the normal rat cerebrum. These findings indicated that postsynaptic markers were surely expressed in hiPSC-neurons at the protein level, in accordance with transcript analysis.

### Evaluation of electrophysiological activity of hiPSC-neurons

**Fig. 5:**
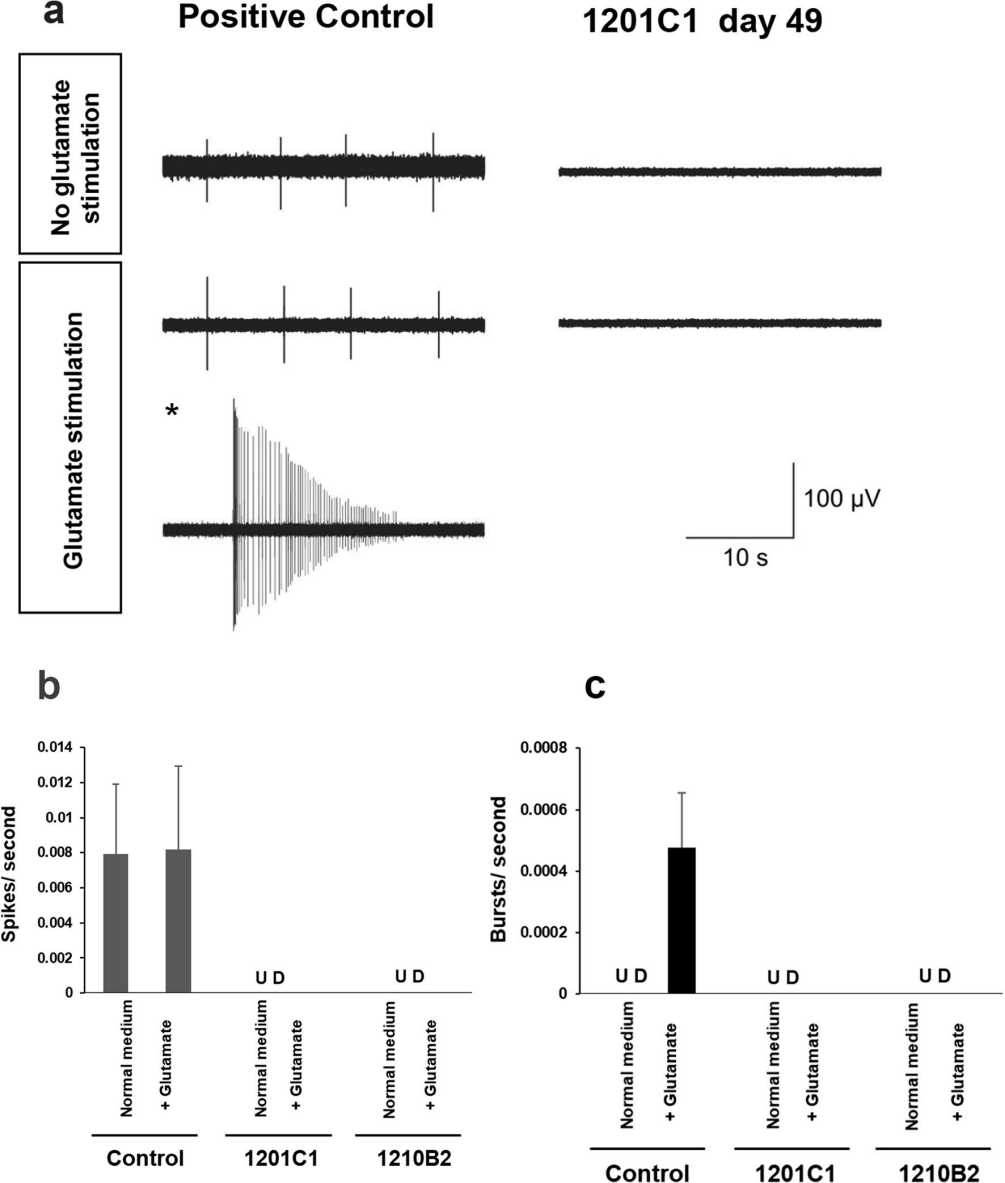
Microelectrode array recording results. **a** Examples of electrophysiological activity are presented. The burst firing seen in the positive control during glutamate stimulation is additionally shown (*). **b**, **c** The graph shows the number of spikes and bursts per second. The bars show the mean, and the error bars show the standard error. Spikes and bursts were detected in the positive control, but no significant spike was detected in the hiPSC-neurons. *UD* undetermined

To examine the neurophysiological maturation of hiPSC-neurons, we analyzed the electrophysiological activity of hiPSC-neurons on day 49 of differentiation using SKY neurons as positive controls. In the positive control, spontaneous spikes were apparently detected in the glutamate-free medium at 21 days, and burst discharges were identified at several measurement points after the addition of 100 μM glutamate, which suggests neurophysiological maturation, although it did not reach a level that showed a significant increase in the total spike frequency (Fig. 5). On the other hand, neither spontaneous spikes nor the response to glutamate stimulation were observed in hiPSC-neurons on day 49 (Fig. 5). These findings indicated that the neurophysiological maturation of hiPSC-neurons was not sufficiently developed on day 49 of differentiation and that they stayed in their immature stage.

### Comparison of pre- and postsynaptic marker gene expression between adult human brain samples and hiPSC-neurons

**Fig. 6:**
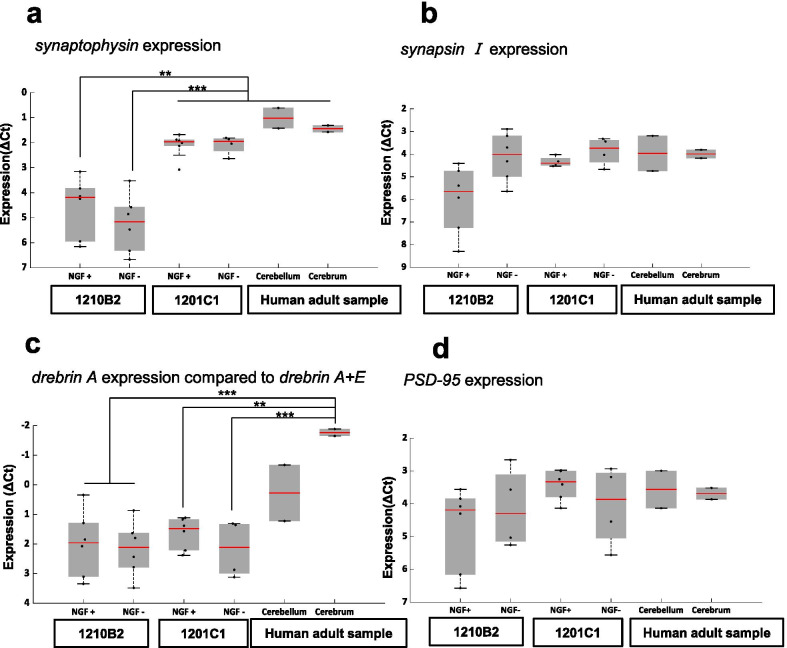
Comparison of synaptophysin and postsynaptic marker expression in hiPSC-NPC-derived neurons on day 49 and human adult brain tissue samples. Dot plots and boxplots showing the gene expression (ΔCt value) on day 49 in hiPSC-derived neurons (1210B2 and 1201C1, both with and without NGF) and adult human brain tissue samples (cerebellum and cerebrum). **a** Presynaptic marker, *synaptophysin*. **b** Presynaptic marker, *Synapsin I*. **c** Postsynaptic marker, *drebrin A* per *drebrinA*+*E*. **d** Postsynaptic marker, *PSD-95*. Statistical analysis was performed using ANOVA with the post hoc Tukey–Kramer method (***p < 0.001, **p < 0.01, *p < 0.05)

On day 49 of differentiation, the gene expression (normalized to *GAPDH*) of the presynaptic marker *SYP* in 1201C1 (but not in 1210B2) cells was similar to that in commercially available adult human cerebellum and cerebrum samples regardless of whether NGF was added (Fig. 6a). Another presynaptic marker, *SYN1*, was expressed in both cell lines at the same level as that in adult brain tissue samples (Fig. 6b). Since there was no significant change in *drebrin A*+*E* mRNA expression over time in our culture and previous reports have shown alternative splicing from *drebrin E* to *drebrin A*, we hypothesized that changes in the drebrin fraction would alter the expression of postsynaptic markers. The expression ratio of *drebrin A* to *drebrin A*+*E* in both the 1210B2 and 1201C1 cell lines tended to be lower than that in the cerebellum samples and significantly lower than that in the cerebrum samples (Fig. 6c). In contrast to *drebrin A*, *PSD-95* expression was the same as that in the adult samples (Fig. 6d). These findings indicate that although hiPSC-neurons generally express pre- and postsynaptic markers at the transcriptional level, the expression levels vary depending on the marker. In particular, *drebrin A* expression does not reach the level of that in adult cerebral tissue.

### Phenotypes of hiPSC-derived neurons after 49 days of differentiation

**Fig. 7:**
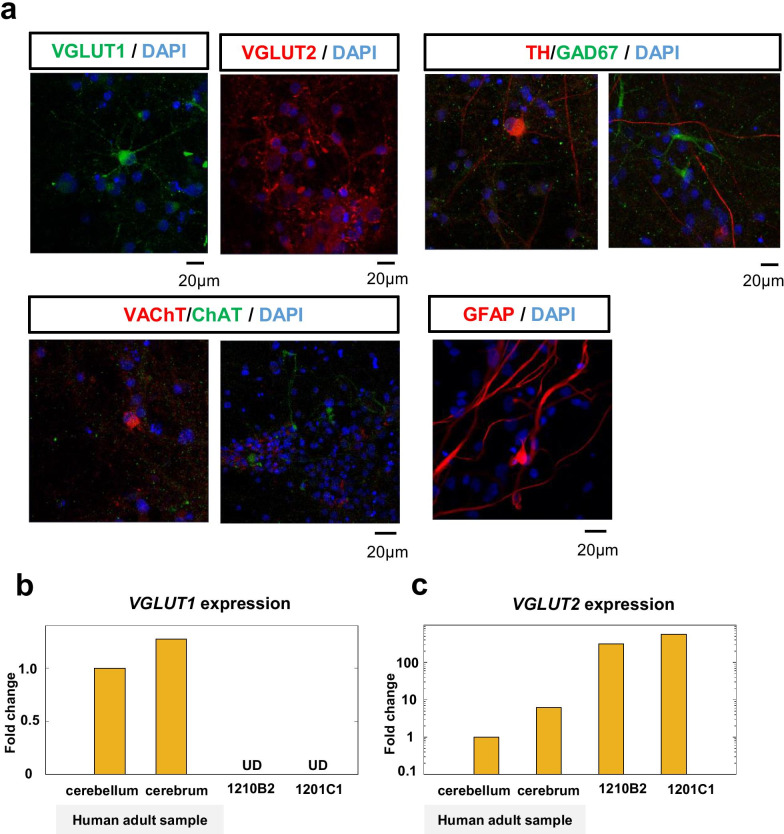
Phenotypic analysis of hiPSC-neurons on day 49 of differentiation. **a** Immunofluorescence analysis of glutamatergic neurons (VGLUT1 and VGLUT2), dopaminergic neurons (TH), GABAergic neurons (GAD67), cholinergic neurons (VAChT and ChAT), and glial cells (GFAP) on day 49 of differentiation in 1210B2 cells. The nuclei were stained with DAPI (blue). Scale bar: 20 μm. **b**, **c** Comparison of the gene expression levels of VGLUT1 (**b**) and VGLUT2 (**c**) in hiPSC-derived neurons (1210B2 and 1201C1, both without NGF) on day 49 and in adult human samples (cerebellum and cerebrum). *UD* undetermined

The number of neurons determined by the ratio of ELAVL3/4-positive cells on day 49 reached 80% or more of the population in two cell lines (1210B2 cell line: NGF+, 88.8%; NGF−, 82.9% and 1201C1 cell line: NGF+, 80.2%; NGF−, 82.8%). Most neurons (> 80%) were VGLUT2-positive glutamatergic neurons (Fig. 7a, Table 1). The number of neurons that were positive for VGLUT1 (another glutamatergic neuronal marker), TH (dopaminergic neuron marker), VAChT, or ChAT (cholinergic neuron marker) was very small (Fig. 7a, Table 1). Additionally, TPH2 (a serotoninergic marker) was not detected in any neurons (Table 1). While few GFAP-positive glial cells were found, many fibers were observed (Fig. 7a). The differential expression patterns of VGLUT1 and VGLUT2 on glutamatergic neurons were confirmed by qRT-PCR analysis (Fig. 7b, c); the expression of VGLUT2 was higher than that in adult human brain tissue samples (Fig. 7c). This result was in contrast to that of VGLUT1 expression (Fig. 7b). These findings showed that hiPSC-NPCs predominantly differentiated into VGLUT2-positive glutamatergic neurons in our culture system.

**Table 1:** Neuronal subtypes analysis of hiPSC-NSPC-derived neurons after 49 days of differentiation

hiPSC neurons	NGF	Type of cells						
		Glutamatergic nerve		Dopaminergic neuron	Serotonergic neuron	Cholinergic neuron		Glial cells
		VGLUT1 positive	VGLUT2 positive	TH positve	TPH2 positve	VAChT positve	ChAT positve	GFAP positive
1210B2	+	+	+++	+	–	+	+	+
	–	+	+++	+	–	+	+	+
1201C1	+	+	+++	+	–	+	+	+
	–	+	+++	+	–	+	+	+

The evaluation of immunofluorescence images are shown (+++: > 80%; ++: 10–80%; +: 1–10%; –: none)

### Multi-institutional validation of the reproducibility of the neural differentiation method

**Fig. 8:**
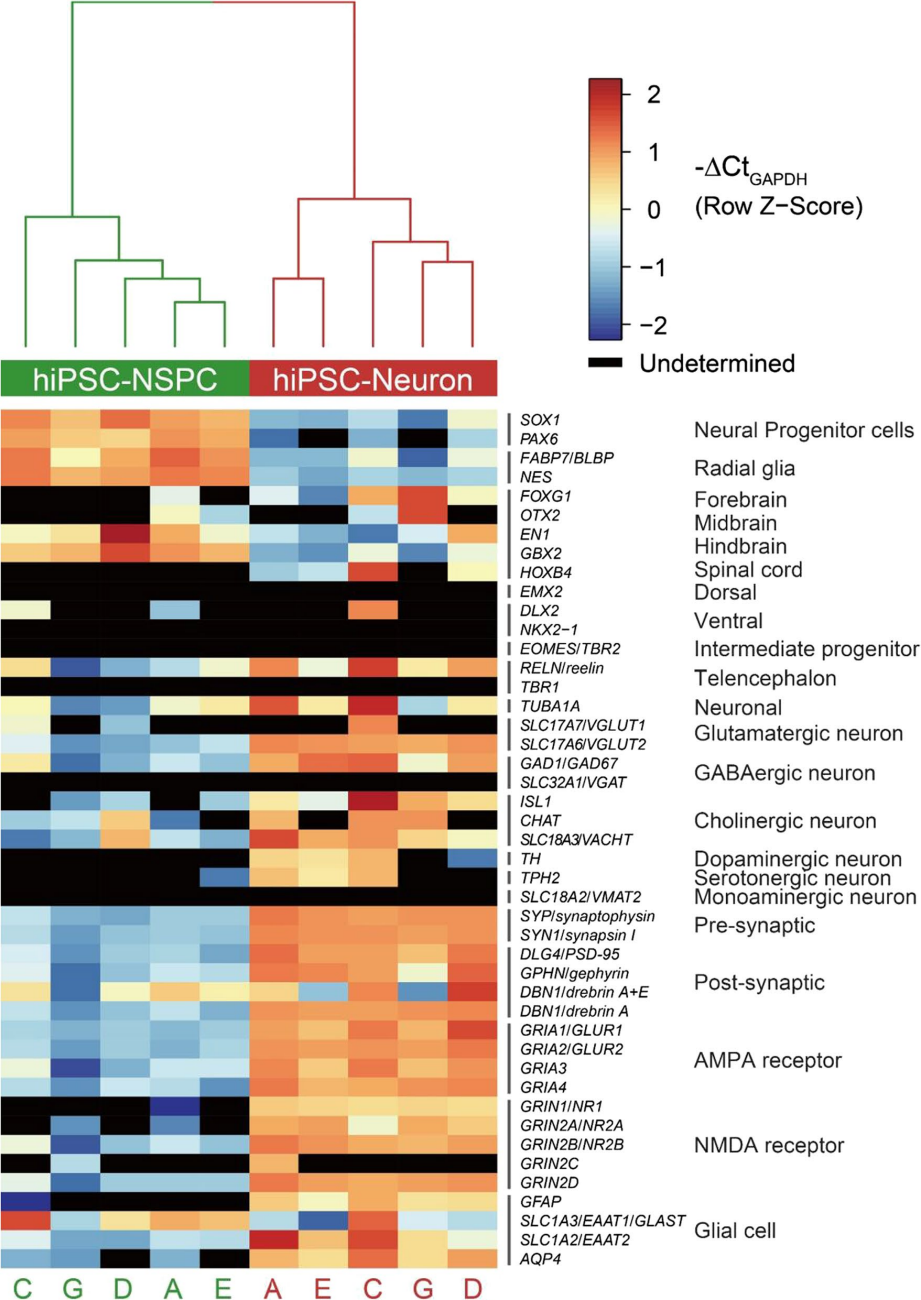
Gene expression analysis of the neural differentiation method at multiple centers. Hierarchical clustering of 1210B2 hiPSC-NPCs and hiPSC-neurons cultured in the absence of NGF (day 49) at multiple centers. A, C, D, and E indicate each center

To evaluate the efficacy and robustness of the differentiation method developed here, the 1210B2 cell line was evaluated at multiple facilities under non-NGF cell culture medium conditions. On days 0 and 49, we performed qRT-PCR analysis to compare the cells that were differentiated at multiple sites (Fig. 8). Similar gene expression patterns were observed in both hiPSC-NPCs on day 0 and hiPSC-neurons on day 49 regardless of the facility at which they were differentiated (Fig. 8). Undifferentiated markers such as *SOX1*, *PAX6*, and *NES* were expressed at low levels, reflecting differentiation. The expression of localization markers such as *EN1* and *GBX2* tended to be lower than that before differentiation. Focusing on the markers of glutamatergic neurons, the expression of *VGLUT1* was not reproducible, but *VGLUT2* expression increased as the cells differentiated. This result was consistent with the immunostaining images presented earlier, which showed a VGLUT2-dominated cell population. Although only a few cells were observed in the stained images, the expression levels of markers of GABAergic neurons, cholinergic neurons, dopaminergic neurons, and serotonergic neurons as well as glial cells were increased, which suggested that the induction of these cell types was advancing. The expression levels of the presynaptic markers *SYP* and *SYN1*, the excitatory postsynaptic markers *PSD-95* and *drebrin*, and the inhibitory postsynaptic marker *gephyrin* as well as those of AMPA and NMDA receptor markers were also confirmed, which suggested progression in the direction of synapse formation. Moreover, approximately 80% (75–87%) of the cell population was ELAVL3/4-positive on day 49 at all facilities.

## Discussion

### Human iPSC-NPCs differentiate into mature neurons expressing neuronal markers and synapse-related proteins, but the expression of postsynaptic markers is incomplete

In this study, we confirmed sufficient maturation of hiPSC-neurons at the level of neuronal phenotypic markers after 49 days of culture. The presynaptic markers *SYP* and *SYN1* were generally well expressed at the mRNA level by day 35, which was generally consistent with the immunostaining images.

However, the postsynaptic markers differed from presynaptic markers. The drebrin E protein was found to be expressed early, whereas the other postsynaptic markers were detected at the level of mRNA transcription, but their protein expression and localization to dendritic spines were insufficient in immunostaining images.

Western blot analysis showed that the protein expression of both presynaptic and postsynaptic markers was equally increased in the day 49 hiPSC-neuron sample compared to the day 0 hiPSC-neuron sample, although the expression was lower compared to the rat brain tissue sample. This suggests that the differences in immunostaining results of pre- and postsynaptic proteins cannot be explained by protein expression alone. The differences in the localization of synaptic proteins may be reflected in the differences in immunostaining results. Functional analysis with MEA recording could not detect significant electrophysiological activity of hiPSC-neurons on day 49. This finding is consistent with previous reports showing that it takes a long time to detect electrophysiological activity in human stem cell-derived neurons [13, 32]. Taken together, these results suggest that the maturation of synaptic structures of hiPSC-neurons was incomplete and that hiPSC-neurons remained immature in their neurophysiological features after 49 days of differentiation.

There are two forms of drebrin, the juvenile form of drebrin E and the mature form of drebrin A, which are generated by alternative splicing from a single gene [17]. During neuronal development, drebrin E is present in growth cones and is involved in axon extension. As neurons mature, drebrin E disappears, and drebrin A appears, accumulating in the postsynaptic region and promoting the formation of dendritic spines [17]. Since drebrin A promotes the accumulation of PSD-95, the conversion of *drebrin E* to *drebrin A* expression is thought to be a useful marker of postsynaptic immaturity and maturation in stages without PSD-95 expression [18, 33]. Focusing on its expression, *drebrin A* was increased for up to 35 days in 1210B2-derived neurons and for up to 49 days in 1201C1-derived neurons. Changes in *drebrin A* mRNA expression were consistent with the trend of changes in the expression levels of other postsynaptic markers, *PSD-95*, *GluR1*, and *NR1*. These findings suggest that our method requires at least 35 days of incubation for dendritic maturation at the mRNA level. However, even at week 7, the protein expression and localization of postsynaptic maturation markers were poor. These results suggest that when examining synaptic maturation, we should be aware that the construction of postsynaptic structures is slower than the differentiation of neurons and presynaptic markers and that their order of expression could be preferable when assessing synapse formation using postsynaptic markers. Drebrin A appears earlier than PSD-95 as a marker of postsynaptic structure maturation during human neuronal differentiation [18, 33]. There is still a time lag between mRNA and protein expression and localization. Additional techniques could be necessary to promote the localization of postsynaptic marker proteins in vitro and to induce early postsynaptic structure maturation.

### In vitro-expanded hiPSC-NPCs predominantly differentiate into VGLUT2-positive glutamatergic neurons

A substantial amount of excitatory synaptic transmission is mediated by glutamate. VGLUTs carry glutamate to synaptic vesicles, and three isoforms have been identified in humans: VGLUT1, VGLUT2, and VGLUT3 [34–36]. Although methods of inducing VGLUT1-positive cells by proliferation at the neural progenitor cell stage have been reported previously [8], there are few similar reports for VGLUT2-positive cells. In this context, the VGLUT2-positive cells derived from hiPSC-NPCs could be a unique finding of our study. VGLUT2-expressing cells have been reported to be located predominantly in the thalamus, hypothalamus (especially the ventromedial nucleus), brainstem, and cortical layer 4 [36], and VGLUT2-positive cells were previously found to be involved in neuropathic pain and central apnea [37, 38]. In the present study, VGLUT1 was not detected by qRT-PCR, and few cells were positive for VGLUT1, as determined by immunocytochemistry staining. These findings could be related to the observation that our hiPSC-NPCs highly expressed a ventral hindbrain marker, which is often associated with VGLUT2-positive cells.

Our hiPSC-NPCs showed significant ventral hindbrain markers, which could have been induced ventrally by the inhibition of bone morphogenetic protein (BMP) signaling during the differentiation induction phase of the dual SMAD inhibition protocol and differentiation into the hindbrain system via the EGF and FGF used in the NPC culture. BMP signaling is known to promote dorsalization during the process of differentiation into neurons, and NPCs are thought to be transformed into hindbrain progenitor cells when EGF and FGF are added [39–41]. However, there have been reports of differentiation into the same ventral side of the brain that differentiates into GAD67-positive cells [9]. Further investigation is needed to determine which factors lead to differentiation into VGLUT-positive cells. In any case, our method reproducibly achieves differentiation into a cell population centered on VGLUT2-positive cells, which could be useful for pathologies that involve VGLUT2-positive cells, such as neuropathic pain and central apnea [37, 38].

### NGF had no significant effect on the differentiation or synapse maturation of hiPSC-NSCs expanded in vitro

NGF is produced in various parts of the central nervous system [19], and the action of NGF on its receptor, tropomyosin receptor kinase A, is particularly important for the survival of cholinergic nerves in the basal forebrain and has been reported to be involved in dendrite formation, at least in cholinergic neurons [20, 21]. Thus, we expected the presence or absence of NGF to alter synaptogenesis and differentiation maturation into cholinergic nerves. However, there was no significant effect on the neuronal subtype or gene expression. These findings suggest that the addition of exogenous NGF does not increase the rate of differentiation into cholinergic neurons, at least not in the early stages of neuronal differentiation, and that NGF might not be associated with the formation of excitatory synapses during their formation.

### The method for differentiating hiPSC-NPCs expanded in vitro into human neurons is somewhat robust and reproducible

The reproducibility of this method was confirmed by a multicenter analysis, and the expression of presynaptic molecules and neural differentiation markers was confirmed with high reproducibility. There was also a trend toward the mRNA expression of postsynaptic markers such as *drebrin* and *GLUR*. This trend suggests that our culture system is a robust method for inducing neural differentiation and excitatory synapse formation regardless of the experimenter. However, several factors can be improved. The method described herein generally requires a long culturing period for the establishment of the most mature postsynaptic structures. To generate mature synaptic structures earlier from human stem cell-derived neurons in vitro, coculture with mature astrocytes or exogenous factors other than NGF in addition to BDNF may be useful. These points should be addressed in future studies.

## Supplementary Information

**Additional file 1: Table 1.** Primers for SYBR Green-based qRT-PCR.

**Additional file 2: Table 2.** Antibodies used in this study.

**Additional file 3: Table 3.** Antibodies used in Western bold analysis.

**Additional file 4:** **Figure S1.** Gene expression analysis of pre- and postsynaptic markers during the neuronal differentiation of hiPSC-NPCs in the absence and presence of NGF. Dot and box plots showing the expression of postsynaptic markers (*drebrin A*+*E* and *drebrin A*) and a presynaptic marker (*synaptophysin*) during neuronal differentiation. Statistical analysis was performed using ANOVA with the post hoc Tukey–Kramer method and Student’s *t*-tests (***p < 0.001, **p < 0.01, *p < 0.05). **Figure S2.** Protein expression of postsynaptic markers in terminally differentiated 1210B2 hiPSC neurons. Time-dependent immunofluorescence images of βIII-tubulin, MAP2, total drebrin, drebrin A, synaptophysin, and PSD-95 in 1210B2 cells in the absence of NGF. The nuclei were stained with DAPI (blue). Scale bar: 20 μm. **Figure S3.** Positive control of immunostaining image. SKY neurons cultured for 21 days were used as a positive control for the PSD-95 antibody. The staining was confirmed by setting up a control. Scale bar: 20 μm. **Figure S4.** Immunostaining images of pre- and postsynaptic markers on day 49 in hiPSC neurons in the presence and absence of NGF. Immunofluorescence images of MAP2, drebrin A, synaptophysin, and PSD-95 in 1210B2 and 1201C1 cells in the presence and absence of NGF on day 49. The nuclei were stained with DAPI (blue). Scale bar: 20 μm. **Figure S5.** Western blot analysis of presynaptic proteins in hiPSC neurons on day 0, day 49, and rat cerebrum tissue lysate. For the rat cerebrum tissue sample, 10 µg of protein per well was used, and for the other samples, 30 µg of protein per well was used for analysis. **Figure S6.** Relative protein expression was assessed by Western blot analysis. The protein expression levels of pre- and postsynaptic marker were determined by the β-Actin expression level used as an internal control and then shown as the relative ratio to the expression level in the control rat cerebrum lysate. *UD* undetermined.

## Data Availability

All data generated and/or analyzed in this study are included in this published article.

## Abbreviations

hiPSC: Human-induced pluripotent stem cell
NPC: Neural progenitor cell
NSPC: Neural stem/progenitor cell
NGF: Nerve growth factor
VGLUT: Vesicular glutamate transporter
qRT-PCR: Quantitative reverse transcription-polymerase chain reaction
ANOVA: Analysis of variance
ChAT: Choline acetyltransferase
EGF: Epidermal growth factor
FGF: Fibroblast growth factor
GAD67: Glutamate decarboxylase 67
GFAP: Glial fibrillary acidic protein
LIF: Leukemia inhibitory factor
MAP2: Microtubule-associated protein 2
PBS: Phosphate-buffered saline
PSD-95: Postsynaptic density protein 95
TH: Tyrosine hydroxylase
TPH2: Tryptophan hydroxylase 2
TrkA: Tropomyosin receptor kinase A
VAChT: Vesicular acetylcholine transporter
