# Supplementary material for: Postsynaptic structure formation of human iPS cell-derived neurons takes longer than presynaptic formation during neural differentiation in vitro
Source: Mol Brain. 2021 Oct 11;14:149. doi: 10.1186/s13041-021-00851-1 (PMC8504131; doi:10.1186/s13041-021-00851-1)
Supplement: Supplementary file 1 — Additional file 1: Table 1. Primers for SYBR Green-based qRT-PCR. [file 13041_2021_851_MOESM1_ESM.docx]

**Additional Table 1 Primers for SYBR Green based quantitative PCR**

| **Primer** | **Forward** | **Reverse** |
| --- | --- | --- |
| ***FOXG1*** | **GCCACAATCTGTCCCTCAACA** | **CGGGTCCAGCATCCAGTAG** |
| ***OTX2*** | **ATCTTCATGCGAGAGGAGGTG** | **CATTCTGCTGTTGTTGCTGTTG** |
| ***EN1*** | **TGGGTGTACTGCACACGTTATTC** | **TGTCCTCCTTCTCGTTCTTCTTCT** |
| ***GBX2*** | **CCAAGTGGAAACGGGTGAA** | **ATGCTGACTTCTGATAGCGAACC** |
| ***HOXB4*** | **ACGTGAGCACGGTAAACCCCAA** | **ATTCCTTCTCCAGCTCCAAGACCT** |
| ***PAX6*** | **ACCTGGCTAGCGAAAAGCAA** | **CCCGTTCAACATCCTTAGTTTATCA** |
| ***PAX7*** | **CAAACACAGCATCGACGGCA** | **GTGAATGTGGTCCGACTGCG** |
| ***NKX2.1*** | **CGCATCCAATCTCAAGGAAT** | **CAGAGTGTGCCCAGAGTGAA** |
| ***NKX2.2*** | **GCTCTGTGGCCGAAGGTCCG** | **GCTTGAGTCCTGAGGGGGCG** |
| ***NKX6.1*** | **TCTTCTGGCCCGGAGTGATG** | **CCCGTCTTTGTCCAACAAAATGGAT** |
| ***EMX2*** | **GCTTCTAAGGCTGGAACACG** | **CCAGCTTCTGCCTTTTGAAC** |
| ***SLC17A7*/*VGLUT1*** | **GAAACTCATGAACCCCCTCA** | **GGGAGATGAGCAGCAGGTAG** |
| ***SLC17A6*/*VGLUT2*** | **ATTCCATCAGCAGCCAGAGT** | **TTGCTCCATATCCCATGACA** |
| ***GAD1*/*GAD67*** | **CGTCTTCGACCCCATCTTCGT** | **CGCAGATCTTGAGCCCCAGTT** |
| ***TH*** | **GTAAGCAGAACGGGGAGGTG** | **GGTACGTCTGGTCTTGGTAGGG** |
| ***TPH2*** | **ATGGCTCAGATCCCCTCTACA** | **GGATCCGCAAGTAGTGGAACA** |
| ***SYP*/*synaptophysin*** | **TCGGCTTTGTGAAGGTGCTGCA** | **TCACTCTCGGTCTTGTTGGCAC** |
| ***DLG4*/*PSD95*** | **TCGGTGACGACCCATCCAT** | **GCACGTCCACTTCATTTACAAAC** |
| ***DBN1*/*drebrin A+E*** | **CACGGAGATCCACGATGCAG** | **TGCCAGGCCATAGGTCAATGAG** |
| ***DBN1*/*drebrin A*** | **TTCATAAAGGCATCGGACAGTGG** | **ATGGGAGGGAGGAAGAGAGGTTTGG** |
| ***GFAP*** | **ACATCGAGATCGCCACCTAC** | **ACATCACATCCTTGTGCTCC** |
| ***GAPDH*** | **CCACTTTGTCAAGCTCATTTCCT** | **TCTCTTCCTCTTGTGCTCTTGCT** |
| ***SOX1*** | **AGCAGTTGTTTCTGGAAGAGTCTGT** | **AGGCCCTTATCCCGGACTAA** |
| ***FABP7*/*BLBP*** | **GGACTCTCAGCACATTCAAGAA** | **CCACATCACCAAAAGTAAGGGT** |
| ***NES*** | **CCAAGACTGCCCTGGAAAC** | **CCTCCCTCTCCAAGGAAACA** |
| ***DLX2*** | **ACGCTCCCTATGGAACCAGTT** | **TCCGAATTTCAGGCTCAAGGT** |
| ***EOMES*/*TBR2*** | **CACCGCCACCAAACTGAGAT** | **CGAACACATTGTAGTGGGCAG** |
| ***RELN*/*reelin*** | **GTAGCAAGCCCTTCAGCAAC** | **CCCTGAGGCCAGTACAACAT** |
| ***TBR1*** | **ATGGGCAGATGGTGGTTTTA** | **GACGGCGATGAACTGAGTCT** |
| ***TUBA1A*** | **AGCTCGGCAGTCGCGAAGCAG** | **CAATGACTGTGGGTTCCAAGTCTAC** |
| ***SLC32A1*/*VGAT*** | **AGATGATGAGAAACAACCCCAG** | **CACGACAAGCCCAAAATCAC** |
| ***ISL1*** | **GTTTGAAATGTGCGGAGTGTAAT** | **TTCTTGCTGAAGCCGATGC** |
| ***CHAT*** | **GGAGGCGTGGAGCTCAGCGACACC** | **CGGGGAGCTCGCTGACGGAGTCTG** |
| ***SLC18A3*/*VACHT*** | **TACGGAGAGCGAAGACGTGAA** | **ATCATGGCTATGCCAGACGTG** |
| ***SLC18A2*/*VMAT2*** | **CGGAAGCTCATCCTGTTCATC** | **CCTGGCCGTCTGGATTTCTG** |
| ***SYN1*/*synapsin I*** | **GACGGAAGGGATCACATCAT** | **CTGGTGGTCACCAATGAGC** |
| ***GPHN*/*gephyrin*** | **CACCAGAATTCGCACTTCAAG** | **AGACAGTAATGCCAGGACAAG** |
| ***GRIA1*/*GLUR1*** | **GGTCTGCCCTGAGAAATCCAG** | **CTCGCCCTTGTCGTACCAC** |
| ***GRIA2*/*GLUR2*** | **AGTTTTCCACTTCGGAGTTCAG** | **CCAAATTGTCGATGTGGGGTG** |
| ***GRIA3*** | **TCCGGGCGGTCTTCTTTTTAG** | **TGGGGAATCCTCCGTGAGAAT** |
| ***GRIA4*** | **GCCATTGTCCCTGATGGAAAA** | **GAGGGGCAATAGCAATCTCTG** |
| ***GRIN1*/*NR1*** | **AGGAACCCCTCGGACAAGTT** | **CCGCACTCTCGTAGTTGTG** |
| ***GRIN2A*/*NR2A*** | **TGGACGTGAACGTGGTAGC** | **CCCCCATGAATGCCCAAGAT** |
| ***GRIN2B*/*NR2B*** | **TTCCGTAATGCTCAACATCATGG** | **TGCTGCGGATCTTGTTTACAAA** |
| ***GRIN2C*** | **GAGTGGTCAAATTCTCCTACGAC** | **TGTAGTACACCTCCCCAATCAT** |
| ***GRIN2D*** | **CTGGCCTCACTGGATCTGG** | **GGAAGGAAACCATAATCACGCA** |
| ***SLC1A3*/*EAAT1*/*GLAST*** | **CGAAGCCATCATGAGACTGGTA** | **TCCCAGCAATCAGGAAGAGAA** |
| ***SLC1A2*/*EAAT2*** | **GGGCACCGCTTCCAGTG** | **ATACTGGCCGCGCCG** |
| ***AQP4*** | **AGCAGTCACAGCGGAATTTCT** | **TCTGTTCCACCCCAGTTGATG** |
