# Supplementary material for: Postsynaptic structure formation of human iPS cell-derived neurons takes longer than presynaptic formation during neural differentiation in vitro
Source: Mol Brain. 2021 Oct 11;14:149. doi: 10.1186/s13041-021-00851-1 (PMC8504131; doi:10.1186/s13041-021-00851-1)
Supplement: Supplementary file 2 — Additional file 2: Table 2. Antibodies used in this study. [file 13041_2021_851_MOESM2_ESM.docx]

**Additional Table 2. The antibodies used in this study**

| **Antibody** | **Manufacture name** | **Manufacturer model number** | **Isotype** | **Dilution ratio** |
| --- | --- | --- | --- | --- |
| **TH** | **Millipore** | **AB152** | **Rabbit IgG poly** | **1:200** |
| **HuC/HuD Clone 16A11** | **Thermo Fisher Scientific** | **A-21271** | **Mouse IgG2b** | **1:100** |
| **VAChT** | **Synaptic Systems** | **139 103** | **Rabbit IgG** | **1:500** |
| **GAD67** | **Millipore** | **MAB5406** | **Mouse IgG2a** | **1:500** |
| **MAP2** | **Millipore** | **AB5622-I** | **Rabbit IgG poly** | **1:500** |
| **Anti-drebrin Clone M2F6** | **Gifted from Shirao Lab at Gunma University** | **hybridoma supernatant** | **Mouse IgG1** | **Undiluted** |
| **Synaptophysin** | **Abcam** | **ab14692** | **Rabbit IgG poly** | **1:200** |
| **VGLUT1** | **Synaptic Systems** | **135 511** | **Mouse IgG1** | **1:100** |
| **VGLUT2** | **Synaptic Systems** | **135 403** | **Rabbit IgG poly** | **1:500** |
| **βⅢtubulin** | **BioLegend** | **801201** | **Mouse IgG2a** | **1:500** |
| **drebrinA** | **IBL** | **28023** | **Rabbit IgG poly** | **1:500** |
| **PSD-95** | **Thermo Fisher Scientific** | **MA1-046** | **Mouse IgG1** | **1:200** |
| **GFAP** | **Abcam** | **ab33922** | **Rabbit monoclonal** | **1:500** |
| **ChAT** | **Atlas antibodies** | **AMAb91130** | **Mouse monoclonal** | **1:1000** |
