# Supplementary material for: Postsynaptic structure formation of human iPS cell-derived neurons takes longer than presynaptic formation during neural differentiation in vitro
Source: Mol Brain. 2021 Oct 11;14:149. doi: 10.1186/s13041-021-00851-1 (PMC8504131; doi:10.1186/s13041-021-00851-1)
Supplement: Supplementary file 3 — Additional file 3: Table 3. Antibodies used in Western bold analysis. [file 13041_2021_851_MOESM3_ESM.docx]

**Additional Table 3. The antibodies used in Western blot analysis**

| **Antibody** | **Manufacture name** | **Manufacturer model number** | **Isotype** | **Dilution ratio** |
| --- | --- | --- | --- | --- |
| **Anti-drebrin Clone M2F6** | **Gifted from Shirao Lab at Gunma University** | **hybridoma supernatant** | **Mouse IgG1** | **1:100** |
| **GluN1/NR1** | **StressMarq Biosciences** | **S308-48** | **Mouse IgG1** | **1:1000** |
| **GluR2** | **GeneTex** | **GTX52359** | **Rabbit IgG poly** | **1:1000** |
| **PSD-95** | **Thermo Fisher Scientific** | **MA1-046** | **Mouse IgG1** | **1:1000** |
| **GluA1/GluR1** | **StressMarq Biosciences** | **SMC-440** | **Mouse IgG1** | **1:1000** |
| **drebrinA** | **IBL** | **28023** | **Rabbit IgG poly** | **1:500** |
| **βⅢtubulin** | **BioLegend** | **801201** | **Mouse IgG2a** | **1:500** |
| **Synaptophysin** | **Abcam** | **ab14692** | **Rabbit IgG poly** | **1:1000** |
| **β-actin** | **Proteintech** | **66009-1-lg** | **Mouse IgG2b** | **1:5000** |
