# Supplementary figures and images for: Postsynaptic structure formation of human iPS cell-derived neurons takes longer than presynaptic formation during neural differentiation in vitro
Source: Mol Brain. 2021 Oct 11;14:149. doi: 10.1186/s13041-021-00851-1 (PMC8504131; doi:10.1186/s13041-021-00851-1)

Fig. S1

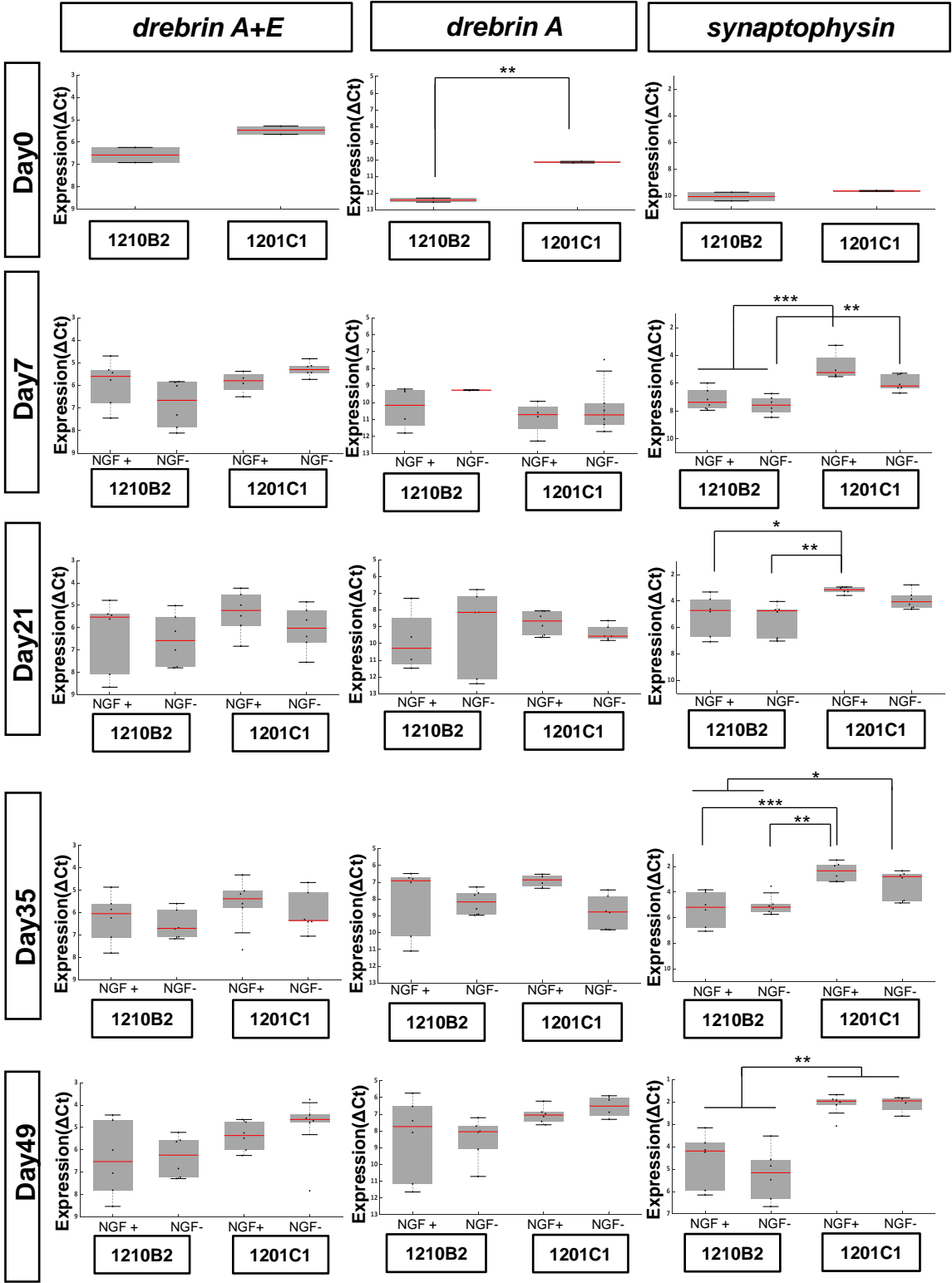

Fig. S2

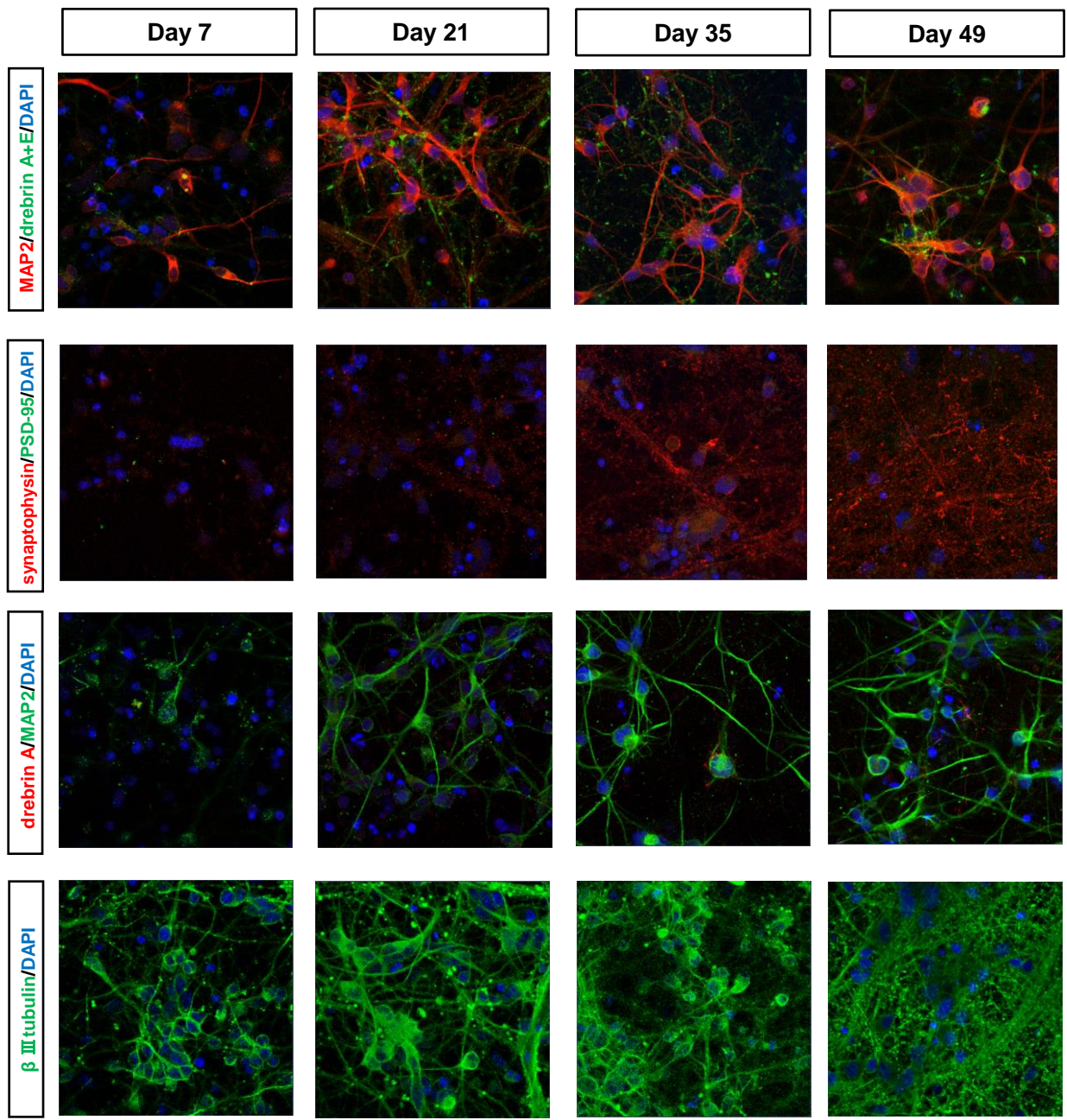

20μm

Fig. S3

MAP2/PSD-95/DAPI

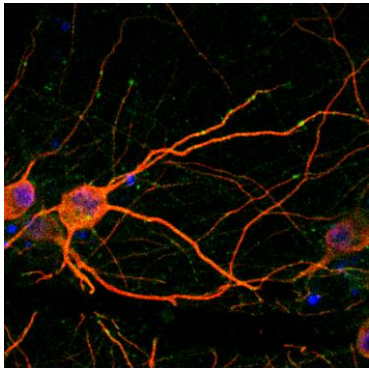

20μm

drebrin A/MAP2/DAPI

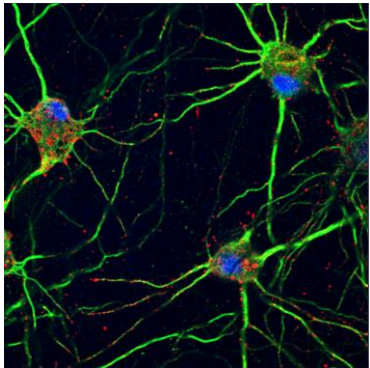

20μm

Fig. S4

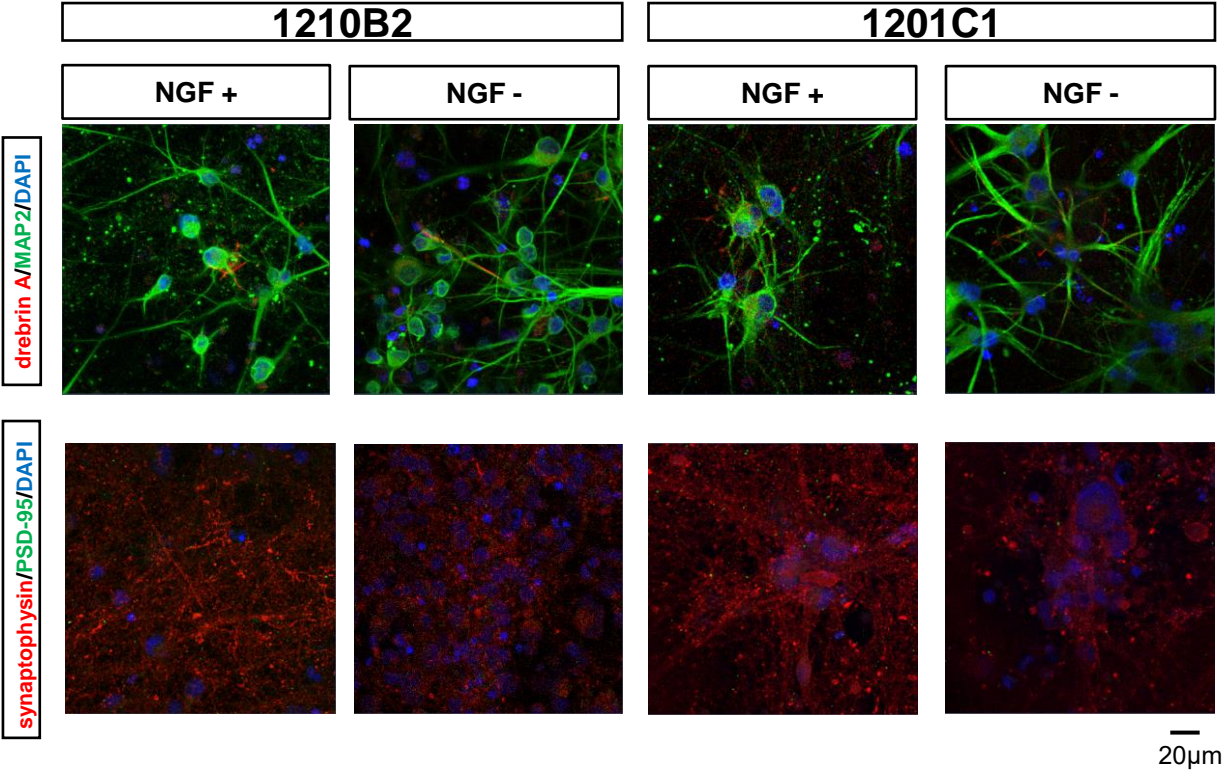

Fig. S5

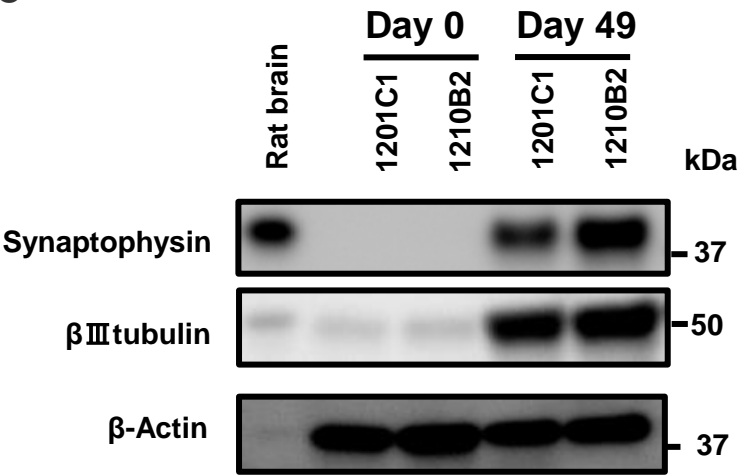

**Fig. S6**

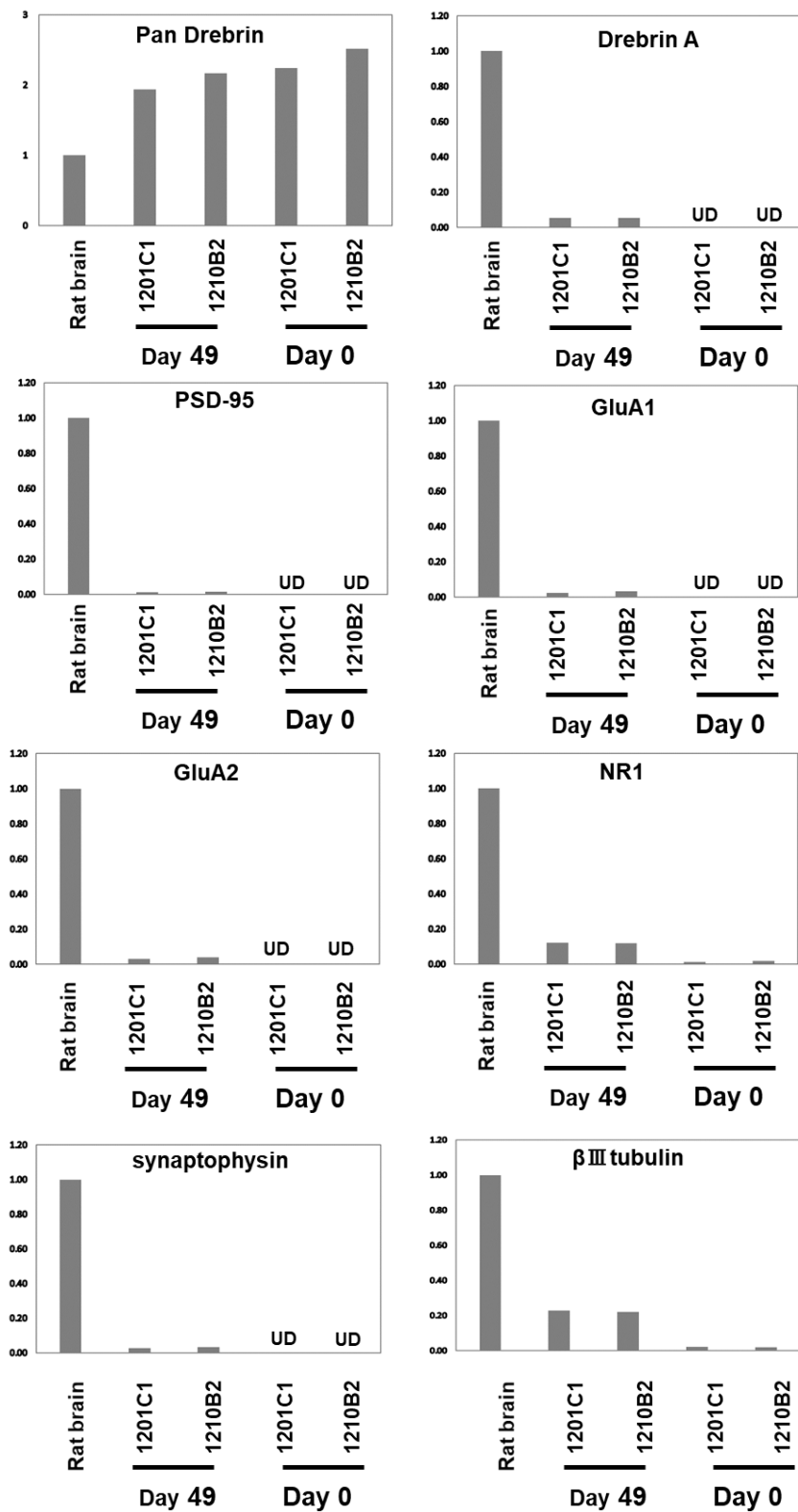

Supplement: Supplementary file 4 — Additional file 4: Figure S1. Gene expression analysis of pre- and postsynaptic markers during the neuronal differentiation of hiPSC-NPCs in the absence and presence of NGF. Dot and box plots showing the expression of postsynaptic markers (drebrin A+E and drebrin A) and a presynaptic marker (synaptophysin) during neuronal differentiation. Statistical analysis was performed using ANOVA with the post hoc Tukey–Kramer method and Student’s t-tests (***p < 0.001, **p < 0.01, *p < 0.05). Figure S2. Protein expression of postsynaptic markers in terminally differentiated 1210B2 hiPSC neurons. Time-dependent immunofluorescence images of βIII-tubulin, MAP2, total drebrin, drebrin A, synaptophysin, and PSD-95 in 1210B2 cells in the absence of NGF. The nuclei were stained with DAPI (blue). Scale bar: 20 μm. Figure S3. Positive control of immunostaining image. SKY neurons cultured for 21 days were used as a positive control for the PSD-95 antibody. The staining was confirmed by setting up a control. Scale bar: 20 μm. Figure S4. Immunostaining images of pre- and postsynaptic markers on day 49 in hiPSC neurons in the presence and absence of NGF. Immunofluorescence images of MAP2, drebrin A, synaptophysin, and PSD-95 in 1210B2 and 1201C1 cells in the presence and absence of NGF on day 49. The nuclei were stained with DAPI (blue). Scale bar: 20 μm. Figure S5. Western blot analysis of presynaptic proteins in hiPSC neurons on day 0, day 49, and rat cerebrum tissue lysate. For the rat cerebrum tissue sample, 10 µg of protein per well was used, and for the other samples, 30 µg of protein per well was used for analysis. Figure S6. Relative protein expression was assessed by Western blot analysis. The protein expression levels of pre- and postsynaptic marker were determined by the β-Actin expression level used as an internal control and then shown as the relative ratio to the expression level in the control rat cerebrum lysate. UD undetermined. [file 13041_2021_851_MOESM4_ESM.pdf]
